# Clinical experience and cost evaluation of magnetic resonance imaging -only workflow in radiation therapy planning of prostate cancer

**DOI:** 10.1016/j.phro.2021.07.004

**Published:** 2021-07-17

**Authors:** Jani Keyriläinen, Olli Sjöblom, Sonja Turnbull-Smith, Taru Hovirinta, Heikki Minn

**Affiliations:** a Department of Medical Physics, Turku University Hospital, Hämeentie 11, FI-20521 Turku, Finland; b Department of Oncology and Radiotherapy, Turku University Hospital, Hämeentie 11, FI-20521 Turku, Finland; c Turku University School of Economics, Information Systems Science, Rehtorinpellonkatu 3, FI-20500 Turku, Finland; d Philips Oy, Philips Medical Systems MR Finland, Radiation Oncology Helsinki, Äyritie 4, FI-01510 Vantaa, Finland; e Department of Finance, The Hospital District of Southwest Finland, Kiinamyllynkatu 4-8, FI-20521 Turku, Finland

**Keywords:** Cost evaluation, MRI-only, Synthetic CT, Radiotherapy planning, Clinical workflow, Prostate cancer

## Abstract

**Background and purpose:**

In radiation therapy (RT), significant improvements have been made recently particularly in the practices of planning imaging. This study aimed to conduct a cost evaluation between magnetic resonance imaging (MRI) -only and combined computed tomography (CT) and MRI workflows.

**Materials and methods:**

The time-driven activity-based costing (TDABC) model was used to conduct a cost evaluation between the two workflows in those steps, where cost differences were expected. Costs were divided into capital costs and operational costs. The former consisted of fixed, one-time expenses, e.g. the purchase of a scanner, whereas the latter were partially based on the amount of activity consumed i.e. time required for image acquisition, image registration and structure contouring.

**Results:**

In a review over a period of 10 years for 300 annual prostate cancer patients, the total cost of the workflow steps included in the study for an individual patient applying the MRI-only workflow was 903 € (100%), comprised of 537 € (59%) capital costs and 366 € (41%) operational costs. The corresponding total cost for an individual patient applying the CT + MRI workflow was 922 € (100%), comprised of 197 € (21%) capital costs and 726 € (79%) operational costs. In 10 years for 3000 patients, a total saving of 58,544 € (2%) was achieved with the MRI-only workflow compared with the dual imaging workflow.

**Conclusions:**

MRI-only workflow is a feasible and economic way to perform clinical RT for localized prostate cancer, in particular for medium- and large-sized departments treating a sufficient number of patients.

## Highlights

• Using MRI-only for prostate cancer radiation therapy planning (RTP) can reduce costs.
• An MRI-only workflow is particularly suitable for medium-sized and large departments.
• Omitting CT in the RTP workflow saves scanner, staff, and patient time.

## 1 Introduction

Prostate cancer is the most common malignant disease among men in the industrialized countries. In 2018, almost 1.3 million new cases occurred, which corresponded to 7% of all cancers and resulted in 359,000 deaths worldwide [1]. In radiation therapy (RT), imaging is a critical part of the workflow since accurate localization of the treatment volume and normal tissues is essential for cure and for avoiding complications. The superior soft-tissue contrast obtainable in magnetic resonance imaging (MRI) enables accurate target and normal structure delineation [2]. The inter-observer variability in defining e.g. the prostate apex is smaller with MRI in comparison to computed tomography (CT) [3]. Today, the widely used practice of using MRI images for radiation therapy planning (RTP) of prostate cancer is based on the co-registration of CT and MRI images. This enables the utilization of additional anatomical details provided by MRI, whereas the dose calculation is based on the electron density information provided by CT. However, the use of two rather than one imaging modality for RTP requires additional work and time. Moreover, the error associated with the co-registration of CT and MRI images and interval changes in organ filling and movement between the two scans increase the uncertainty in treatment accuracy [3]. For these reasons, it would be ideal to create an RTP practice, which is based on a single imaging modality only. Since 2017, we have implemented an MRI-only workflow by obtaining geometrically and dosimetrically accurate synthetic CT (sCT) images generated from MRI images [4], [5], [6]. All the individual steps in this workflow have been carefully tested and validated before they were implemented into a routine clinical workflow [7], [8], [9], [10].

However, the economic assessment of the real costs of health care, recognized as increasingly important in sound decision-making on the allocation of limited societal resources, has not been carefully examined [11], [12]. Since the share of the expenditures committed in health care is a substantial part of the gross domestic product of nations, a better understanding of the factors contributing to the costs of health care including the costs of medical innovations on a detailed level is urgently needed [12], [13], [14]. Conducting a cost evaluation of an MRI-only practice is important, as the costs of oncologic care are increasing [15]. Various models have been proposed to calculate costing in RT, including that adapted for RT from the activity-based costing (ABC) model in Leuven [16], [17]. When applying this method, the cost of a ‘product’ is calculated. In this context, the product is a course of RT, including its care process activities, e.g. RTP or RT delivery, and the related resources [18].

A newer version of the ABC model – time-driven activity-based costing (TDABC), was developed based on the principles of the ABC model using time as the unique cost driver [18], [19], [20], [21], [22]. It can be defined as a bottom-up method, determining the costs step-by-step of all the resources allocated for each of the activities, e.g. personnel, material, equipment and facilities. The TDABC covers all costs during the entire patient treatment process [22]. The costs are determined by estimating the cost per time unit of the supplying resource capacity and observing the time the resources are committed to specific activities [18]. Using this method, more accurate and transparent estimates of the real expenses incurred by the providers can be achieved [22]. TDABC calculates precisely the actual expenses of the real use of the allocated resources over the entire therapeutic cycle such as RT of a patient with prostate cancer, as chosen for this study [14]. So far, the model remains relatively unexplored in prostate cancer RT and thus, our approach provides a novel insight into the subject.

We present here our clinical experience and a health economy aspect of a recent implementation of an MRI-only workflow for RTP of prostate cancer. The aim of the work was to conduct a cost evaluation between MRI-only and combined CT + MRI workflows. Under the current rising cost pressures of oncologic treatment, it is important to study alternative approaches, which do not compromise quality but balance increased spending for novel biologic drugs.

## 2 Materials and methods

### 2.1 Patients

No ethics approval was required for this study. As per our routine clinical practice at the Department of Oncology and Radiotherapy of Turku University Hospital (TUH, Turku, Finland), patients with localized prostate cancer are treated with RT using three distinct fractionation schedules. The conventional schedule comprised of 37–39 fractions to 74–78 Gy over 7–8 weeks. A mildly hypofractionated schedule was 20 fractions to 60 Gy over 4 weeks and ultrahypofractionated schedule 5 fractions to 36.25 Gy within 11 days or once a week [23]. By February 2021, almost 850 MRI-only-based prostate RT plans have been created for treatment.

Fig. 1a shows the MRI-only workflow in external beam RT, where patients were planned with the MRI-only procedure without the use of planning CT. Fig. 1b presents the combined CT + MRI workflow in external beam RT, where patients were planned utilizing both MRI and CT images: MRI images were co-registered to CT for target delineation and CT was used as a basis for dose calculation. Daily image guidance (IG) with either two-dimensional (2D) kV-radiography or three-dimensional (3D) cone-beam CT (CBCT) was applied for all patients. Both workflows are described in detail in Supplementary material, and Fig. 2 shows the patient setup.

**Fig. 1a:**
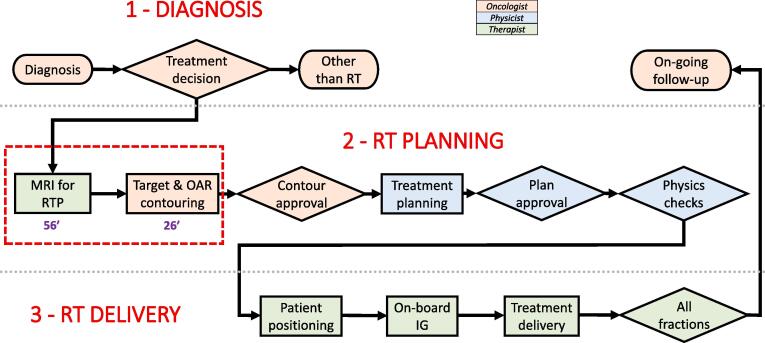
Magnetic resonance imaging (MRI) -only workflow in the external beam radiation therapy (RT) of prostate cancer. The study includes operational costs only for the workflow steps, where cost differences were expected, as marked with a red dashed line. The mean times the personnel spent in completing the MRI for RTP and structure contouring were 56 (SD 5) min and 26 (SD 7) min, respectively (RTP: radiation therapy planning, SD: standard deviation, OAR: organs at risk, IG: image guidance; modified from [34]). (For interpretation of the references to color in this figure legend, the reader is referred to the web version of this article.)

**Fig. 1b:**
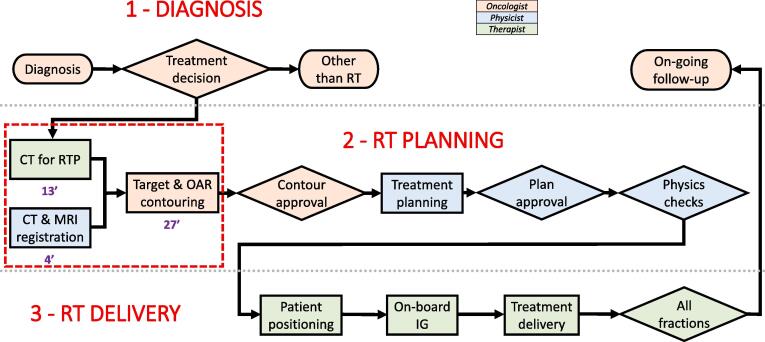
Computed tomography (CT) + magnetic resonance imaging (MRI) workflow in the external beam radiation therapy (RT) of prostate cancer. Note that the MRI examination has been performed externally, so there were no activities based on time at the Department of Oncology and Radiotherapy. The study includes operational costs only for the workflow steps, where cost differences were expected, as marked with a red dashed line. The mean times the personnel spent in completing the CT for RTP, image registration and structure contouring were 13 (SD 4) min, 4 (SD 1) min and 27 (SD 11) min, respectively (RTP: radiation therapy planning, SD: standard deviation, OAR: organs at risk, IG: image guidance; modified from [34]). (For interpretation of the references to color in this figure legend, the reader is referred to the web version of this article.)

**Fig. 2:**
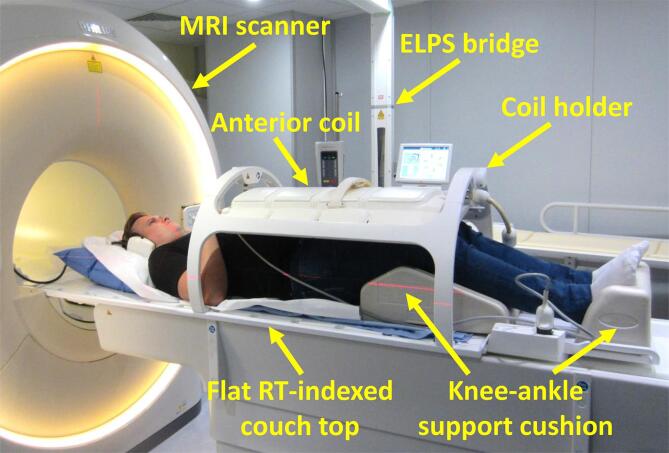
Patient setup on the magnetic resonance imaging (MRI) scanner used for the radiation therapy (RT) planning of prostate cancer: an external laser positioning system (ELPS), an anterior coil placed above the patient using a coil holder, a flat RT-indexed couch top and a knee-ankle support cushion.

### 2.2 Time-driven activity-based costing

In general, costs can be divided into capital costs and operational costs [24]. Capital costs are fixed, one-time expenses incurred on the purchase of buildings, construction, and equipment used in the production of goods or in providing services. Operational costs can include all expenses related to running an RT department, such as salaries, consumables, maintenance, amortization and overhead expenses, e.g. electricity and cleaning. The cost per patient treated is obtained by dividing the sum of capital and operating costs through a lifetime period by the number of patients treated during the same period.

To calculate the costs based on the TDABC model, workflow diagrams encompassing the steps from diagnosis to follow-up were created for the MRI-only and CT + MRI workflows. The CT + MRI workflow of the study represents the situation at the Department of Oncology and Radiotherapy of TUH prior to the decision to acquire a dedicated MRI simulator to the department. Thus, in the CT + MRI workflow of the study, MRI examinations were purchased externally. Personnel resources, i.e. radiation oncologists (RO), physicists and radiation therapists (RTT), were allocated to each of the workflow steps, as indicated by color coding in Fig. 1. The MRI-only and CT + MRI workflows were compared and the steps differing between the workflows, i.e. imaging for RTP, image registration, and structure contouring, were selected for further assessment. For the afore-mentioned steps, the time the personnel spent in completing the steps was determined. The durations of imaging for RTP were determined by timing 10 MRI and 12 CT sessions for RTP, respectively and, the duration of image registrations was determined by timing 10 cases. The durations of structure contourings on CT and MRI images were obtained from our previous studies [10], [25]. The capacity cost rate (CCR) was determined for all the personnel resources used in the process. The CCRs were based on the mean annual salary costs and the mean annual working hours of the TUH employees in three above-mentioned occupational groups. The CCR for each occupational group was obtained by dividing the annual salary costs by the annual working time of the occupational group. For the sake of simplicity, overhead expenses, e.g. electricity and cleaning, were ignored in this study. The costs of the selected workflow steps were calculated by multiplying the mean time spent in completing the step by the capacity cost rate of the personnel resource used in the step.

In addition to the costs of the personnel resources, the following costs were included in the study: the purchasing of MRI and CT scanners, renovation of the scanner rooms, annual maintenance of the scanners, as well as the costs of MRI coil and CT X-ray tube replacements. The prices of the scanners and their maintenance contracts were average prices based on seven recent offers from different vendors at various hospitals in Finland. For calculating the granulations of the annual costs, the annual number of prostate cancer patients was 300, which is the current capacity at TUH. Different patient volumes, as well as other site-specific characteristics, can be investigated using the spreadsheet file in the Supplementary material. For the long-term expense items, such as purchasing imaging devices and the renovation expenses of an existing old CT scanner room, a write-off period of 10 years was used. Furthermore, the cost of an MRI examination purchased externally was included in the costs of the CT + MRI workflow. The total costs of the steps of the MRI-only and CT + MRI workflows included in the study were calculated over a period of 10 years for 300 annual prostate cancer patients.

## 3 Results

Over a period of 10 years for 300 annual prostate cancer patients, the total cost of the workflow steps studied for an individual patient applying the MRI-only workflow was 903 € (100.0%), comprising of 537 € (59.4%) capital costs and 366 € (40.6%) operational costs. The single most expensive component was purchasing the MRI scanner: 470 € (52.0%). The 903 € total cost per patient comprised of 44 € (4.9%) personnel costs and 859 € (95.1%) space and equipment costs (see Table 1). The total decennial cost of the MRI-only workflow for 300 annual prostate cancer patients for the steps included in the study was 2,708,203 € (100.0%) consisting of 1,609,513 € (59.4%) capital costs and 1,098,690 € (40.6%) operational costs.

**Table 1:** Overview of the costs of the MRI-only and CT + MRI workflows for the steps included in the study over a period of 10 years for 300 annual prostate cancer patients. In the MRI-only workflow, the space and equipment costs included the long-term investments, purchasing the MRI equipment 470 € (52.0%) and room renovation required for installing the equipment 67 € (7.4%), as well as the annual costs for the equipment maintenance contract 239 € (26.4%) and replacement of an MRI coil once in a year 83 € (9.2%). The personnel costs included 56 (SD 5) min image acquisitions by RTTs, i.e. 24 € and 26 (SD 7) min structure contourings by ROs, i.e. 20 €. In the CT + MRI workflow, the space and equipment costs included the long-term investments, purchasing the CT equipment 173 € (18.8%) and room renovation required for installing the equipment 23 € (2.5%), as well as the annual costs for the equipment maintenance contract 268 € (29.1%) and replacement of an X-ray tube once in three years 108 € (11.7%). The personnel costs consisted of 13 (SD 4) min image acquisitions by RTTs, i.e. 6 €, 4 (SD 1) min image registrations by physicists, i.e. 2 € and 27 (SD 11) min structure contourings by ROs, i.e. 21 €. The MRI examinations were purchased externally i.e. from the Department of Radiology of TUH using 320 € (34.7%) per patient, so there were no activities based on time at the RT department. The MRI examinations could also be purchased from the private sector, the cost being up to 672 € depending on the service provider. All costs are VAT 0% (SD: standard deviation, VAT: value added tax, MRI: magnetic resonance imaging, CT: computed tomography, RTT: radiation therapists, RO: radiation oncologist).

Cost components	MRI-Only Workflow		CT + MRI Workflow	
	Cost	Cost per patient	Cost	Cost per patient
**Capital Costs**				
**One-time fixed costs**				
Basic assembly of a scanner without options	1 409 513 €	470 €	519 857 €	173 €
Renovation of a scanner room	200 000 €	67 €	70 000 €	23 €

**Total decennial capital cost**	1 609 513 €	537 €	589 857 €	197 €

**Operational costs**				

**Maintenance and material costs**				
Annual maintenances of a scanner	71 603 €	239 €	80 414 €	268 €
MRI coil replacements (1 pcs/y)	25 000 €	83 €	0 €	0 €
CT’s X-ray tube replacements (1 pcs/3 y)	0 €	0 €	32 500 €	108 €
**Personnel costs by workflow step**				
Annual external MRI examinations	0 €	0 €	96 000 €	320 €
Annual image acquisitions by RTTs	7 236 €	24 €	1 734 €	6 €
Annual image registrations by physicists	0 €	0 €	735 €	2 €
Annual structure contourings by ROs	6 030 €	20 €	6 306 €	21 €

**Total annual operational cost**	109 869 €	366 €	217 689 €	726 €

**Total decennial cost**	2 708 203 €	903 €	2 766 747 €	922 €

The corresponding total cost for an individual patient applying the CT + MRI workflow (the workflow steps studied) was 922 € (100.0%), comprising of 197 € (21.3%) capital costs and 726 € (78.7%) operational costs. The single most expensive component was an MRI examination purchased externally: 320 € (34.7%). The 922 € total expense per patient comprised of 29 € (3.2%) personnel costs and 573 € (62.1%) space and equipment costs (see Table 1). The total decennial cost of the CT + MRI workflow for 300 annual prostate cancer patients for the steps included in the study was 2,766,747 € (100.0%) consisting of 589,857 € (21.3%) capital costs and 2,176,890 € (78.7%) operational costs. Thus, in 10 years for 3000 patients, a total saving of 58,544 € (2.1%) was achieved with the MRI-only workflow compared with the standard dual imaging workflow.

**Fig. 3:**
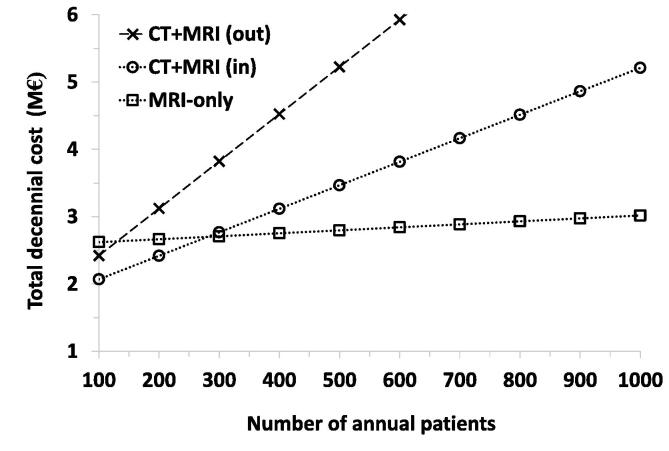
Total decennial cost as a function of the number of patients per year for the MRI-only and CT + MRI workflows, where “out” stands for an external purchase from outside the hospital (672 €/examination) and “in” stands for an external purchase from inside the hospital (320 €/examination). In the latter, the total cost becomes similar if there are 281 patients annually (2,699,801 € and 2,700,390 € for the MRI-only and CT + MRI workflows, respectively). One may note that the higher the number of annual patients is, the higher the savings are in the MRI-only group in comparison to the standard dual imaging workflow, where the MRI examination is purchased externally. For example, if the number of annual prostate cancer patients is 500 or 1000, the total cost is 668,604 € or 2,193,754 € lower in the MRI-only group. On the other hand, for e.g. 100 or 200 annual prostate cancer patients, the total cost is 551,516 € or 246,486 € higher in the MRI-only group than in the dual imaging group. Additionally, if the MRI examination is purchased from outside the hospital for 672 €, the total cost becomes similar already with as low as 130 annual prostate cancer patients (2,633,029 € and 2,630,622 € for the MRI-only and CT + MRI workflows, respectively) (CT: computed tomography, MRI: magnetic resonance imaging).

An overview of the costs in the MRI-only and combined CT + MRI workflows is shown in Table 1, and Fig. 3 shows the total decennial cost as a function of patients per year in the MRI-only and CT + MRI workflows.

## 4 Discussion

The goal of applying an MRI-only workflow to RTP is to remove the planning CT from the workflow and thereby eliminate the registration uncertainty and save resources compared with applying both CT and MRI. Worldwide the method, however, has still been implemented in quite a small number of RT departments. Currently at TUH, the MRI-only workflow has mainly replaced the routine clinical workflow for patients receiving definitive treatment for prostate cancer, and we describe here the economic consequences of implementing it.

Several articles concerning the calculation of health care costs have been published. For instance, Keel et al. [20] have conducted an extensive literature review on the application of the TDABC model in health care. The TDABC model applied here utilizes straightforward entry data and should be easily applicable in different departments and countries but naturally, the figures vary from department to department. Thus, we need to stress the fact that the results in this study are significant only for Finland but the methodology may be applicable also to other countries. Furthermore, here we have actually under-estimated the cost saving attributable to the MRI-only workflow due to the fact that the full cost of purchasing the MRI and CT scanners was attributed to prostate cancer patients alone, i.e. the other patient groups that would also benefit from these scanners were not taken into account. Therefore, in the Supplementary material, there is an additional spreadsheet file, where this issue has been taken into account. The file can also be used for cost evaluation in different RT departments and countries having input data different from those used in this study.

In the operational costs of the current study, particularly the personnel costs are minor for both workflows, representing only 3–5% of the total cost. A similar observation can be made about the capital costs of renovations, which are 2–7% of the total cost for both workflows. In the MRI-only workflow, an estimated room renovation cost of 200,000 € was based on the renewal of a cooling device and network, the renewal of an electricity switchboard and a supply cable, the removal of surface floor (radiofrequency cage) and the installation of a 20-m long gas emission pipeline (500 €/m). In the CT + MR workflow, an estimated room renovation cost of 70,000 € was based on the renewal of a cooling device and electricity as well as the redecoration of the room. Table 1 and Fig. 3 provide a comprehensive view of the expenses and their distribution. In the operational costs, the scanner’s maintenance costs are similar (36–41%) for both workflows. Although the capital costs of the MRI-only workflow are approximately 2.7 times those of the CT + MRI workflow, the benefit of applying the MRI-only method begins to increase as the annual number of patients grows. On the other hand, already with the present annual volume of 300 patients, the operational costs of MRI-only workflow are only 50% of those of the CT + MRI workflow. This is very strongly due to the fact that in the latter, the costs of MRI examinations purchased externally represent up to 35% of the total cost. Additionally, the results of cost evaluation between the two workflows also depend on where the MRI examinations are purchased, varying between 320 € and 672 € per examination at TUH (Fig. 3). In further research, it would be interesting and possibly important to conduct a sensitivity analysis to determine what the drivers of cost and therefore results are.

In addition to the economic savings described above, moving to an MRI-only workflow has obvious clinical advantages. First, the MRI images are obtained in the treatment position, which results in more precise structure contouring and excludes inherent problems in the image co-registration, which is subject to errors associated with registration itself and temporal changes in organ filling and movement between the two scans. Our clinical experience of the MRI-only workflow with almost 1000 treated patients has been collected in selected patient groups with pelvic-region tumors. These first included patients receiving definitive and post-operative prostate cancer RT, later followed by rectal, bladder and gynecological cancer excluding those requiring irradiation of the para-aortic regions. Second, omitting CT considerably saves both scanner and staff time. Moreover, each patient saves one acquisition procedure including additional waiting time and possible accompanying travel costs. Further savings and re-allocation of staff resources are possible through the utilization of automated image segmentation tools for contouring, this task in general being quite highly time and resource intensive [10], [25], [26], [27]. This enables creating the standard anatomical structures required for RTP in parallel with the image acquisition. We underline that all these savings need to be confirmed in prospective studies evaluating clinical outcome and quality of life in the long-term. Third, the exposure to ionizing radiation for diagnostics is reduced, although its significance is negligible compared with the later exposure of ionizing radiation for therapy. The ability to utilize MRI images as the primary reference images for IG also enables sufficient accurate patient positioning during RT delivery [8].

According to our clinical experience, the MRI-only workflow can be successfully accomplished for most prostate cancer patients. Contraindications or other reasons to use CT, such as technical difficulties, accounted for <8% of the cases. This is in good agreement with the data from other clinics: several authors have assessed that MRI-only protocol suffices for safe and effective RT in 88–95% of their prostate cancer patients [28], [29], [30]. It is not very straightforward to include the potential impact on costs of having to use a CT scanner for 5–12% of MRI-only patients in the TDABC model. However, in the foreseeable future, CT will still have an important role at least in RTP of lung and breast cancers. Therefore, a CT scanner is not only a complementary but also a mandatory device at the RT department and thus, the impact on costs of maintaining a CT-based back-up workflow for 5–12% of MRI-only patients is to be considered as insignificant.

We have also verified that the dosimetric agreement between the CT and sCT plans is within 0.5% for all structures [8], which is similar to those published elsewhere [29], [31]. Recently, in addition to MRI-based RTP there has been considerable development in hybrid MRI treatment devices, such as MRI-cobalt and MRI-linear accelerator (linac) systems [32], [33]. These enable real-time MRI-guidance and on-line adaptive planning that will most probably increase the clinical interest to MRI-only RTP. A department considering MRI-only RTP, however, should evaluate the usefulness of the entire process since the possible advantages of MRI-only RTP could be suppressed by disadvantages if the workflow is not fast, accurate and resource-saving [30]. Although there may be an increase of applying an MRI-only workflow in the near future, CT will still play an important role due to its geometric accuracy, quickness and wide accessibility. Other reasons for retaining the standard CT are patient obesity, inability to lie motionless during acquisition and implants, which all may introduce artifacts and geometrical distortions.

In conclusion, our clinical experience and data indicate that an MRI-only workflow is a feasible and economic way to perform clinical RTP for localized prostate cancer, in particular for medium- and large-sized departments with a sufficient number of patients and trained staff adapted to special challenges associated with MRI rather than CT-based RTP. The most important limitation of an MRI-only workflow is the inability to omit CT in RTP of selected patients due to their intercurrent conditions, movement during image acquisition or certain implants. Some of these problems may be overcome by the development of metal artifact reduction MRI sequences and by the increase of the field of view in dedicated MRI scanners.

## Funding

This work was supported by the State Research Funding: the expert responsibility area of 10.13039/501100011797Turku University Hospital, Finland (project no. 13317). The funding had no role in: study design; in the collection, analysis and interpretation of data; in the writing of the report; nor in the decision to submit the article for publication.

## Declaration of competing interest

The authors declare the following financial interests/personal relationships which may be considered as potential competing interests: Sonja Turnbull-Smith, M.Sc. (Tech.), M.D., is employed by Philips Oy, Philips Medical Systems MR Finland, Radiation Oncology Helsinki (Vantaa, Finland). In addition, Philips Oy and Turku University Hospital (Turku, Finland) have signed a master research agreement.
