# Supplementary material for: Clinical experience and cost evaluation of magnetic resonance imaging -only workflow in radiation therapy planning of prostate cancer
Source: Phys Imaging Radiat Oncol. 2021 Jul 17;19:66–71. doi: 10.1016/j.phro.2021.07.004 (PMC8295845; doi:10.1016/j.phro.2021.07.004)
Supplement: Supplementary data 1 [file mmc1.docx]

Clinical experience and cost evaluation of magnetic resonance imaging -only workflow in radiation therapy planning of prostate cancer

**Supplementary material**

***Imaging for radiation therapy planning***

For all patients prior to imaging for RTP, either three nitinol (nickel titanium naval ordnance laboratory) fiducial markers 5.0 mm in length and 1.0 mm in diameter (Beampoint AB, Sollentuna, Sweden) or two gold fiducial markers 10.0 mm in length and 0.4 mm in diameter (Gold Anchor, Naslund Medical AB, Huddinge, Sweden) were routinely implanted into the prostate under ultrasound guidance. This was carried out by urologists at least two weeks before RT. Markers were used to confirm and monitor the prostate position before and during RT using IG.

Today at TUH, the MRI-only workflow is routinely applied to external beam RTP of almost all pelvic cancers. In the evening before the MRI, all prostate cancer patients used rectal enema (Microlax, Delpharm Orléans SAS, Orléans, France). The scheduled length for an appointment in the MRI unit at RT department was 60 min. One to two hours before MRI, patients were instructed to void and then drink 2-4 dl of water. MRI data acquisition was performed by RTTs using a 1.5 T wide-bore (70 cm) MRI scanner (Philips Ingenia 1.5 T HP, Philips MR Medical Systems International B.V., Best, Eindhoven, The Netherlands). The dedicated MRI-RTP platform includes a flat RT-indexed couch top and an external laser positioning system (ELPS, LAP GmbH Laser Applikationen, Lüneburg, Germany) for patient positioning. In pelvic image acquisition, the scanner-integrated body and posterior coils are applied together with an anterior coil placed above the patient using a coil holder to prevent body outline deformation. This setup on MRI matches that on CT, where a knee-ankle support cushion (Civco Radiotherapy, Orange City, IA, USA) and a flat RT-indexed couch top are used, similarly as on treatment units.

Four imaging sequences for RTP were acquired with the patient in a supine position in the following order: 1) T1-weighted 3D mDIXON images with a spatial resolution of 1.14 × 1.14 × 1.14 mm^3^ for the generation of the sCT image applied to dose calculation and a reference for IG, 2) T2-weighted 3D turbo spin echo (TSE) images for target and anatomy delineation, 3) T2-weighted 2D TSE images for target and anatomy delineation, and 4) T1/T2-weighted 3D balanced fast field echo (bFFE) images for marker visualization and localization. These images were sent by RTTs to the RTP system (Eclipse™ versions 13.6 and 15.6, Varian Medical Systems Finland Oy, Helsinki, Finland). Regularly, additional sequences were recorded for diagnostic purposes, e.g. for the visualization of the lymph nodes. A detailed description of the used methods and sequences has been presented in our earlier studies [7-10]. The mean (standard deviation, SD) image acquisition time was 56 (5) min, which is based on the measured time between patient’s arrival to and patient’s departure from the MRI scanner room. An acquisition of the MRI sequences used for RTP took about 13 min and the additional diagnostic MRI sequences took another 13 min. No endorectal coil or gadolinium contrast was used.

The patients underwent the routine CT for RTP if one or more of the exclusion criteria for the MRI-only approach was fulfilled. These include uncertainties of fiducial marker identification, patient movement, patient obesity or metal objects, e.g. hip prostheses, in the patient body. Both MRI and CT scans were always performed for the RTP of ultrahypofractionated stereotactic body radiation therapy (SBRT) treatments of prostate [23]. Prior to the CT image acquisition, patients followed the above-mentioned instructions for bladder and bowel emptying as explained for MRI. The appointment in the CT unit at RT department was scheduled for 30 min. CT images were obtained by RTTs using a whole-body wide-bore (90 cm) CT scanner (Toshiba Aquilion LB, Toshiba Corp., Tokyo, Japan) together with a flat RT-indexed couch top, ELPS and a knee-ankle support cushion. After recording anterior and lateral scanograms, trans-axial images were acquired with reconstruction’s slice thickness of 2.0 mm (1.0 mm in SBRT), 1.0 × 1.0 mm^2^ in-plane spatial resolution, 120 kV tube voltage and tube current modulation in cranio-caudal direction (Toshiba Sure-Exposure 3D SD 12.50). The CT scans were acquired in the supine position and the mean acquisition time was 13 (4) min, which is based on the measured time between patient’s arrival to and patient’s departure from the CT scanner room. In a dual imaging workflow, the CT images were rigidly registered to the MRI images with the aid of fiducial markers. The mean time for manual CT and MRI co-registration by physicists was 4 (1) min.

***Target and organs at risk contouring***

Manual structure contouring of target and organs at risk (OAR) was performed using the Eclipse™ RTP system. After automated boundary detection of body outline, prostate, seminal vesicles, CTV, bladder, rectum, and penile bulb (also urethra in SBRT) were delineated by ROs. Femoral heads, supporting structures for optimization and outlines of implanted fiducial markers were defined as virtual markers (a structure together with line end points) by the planners i.e. either physicists or RTTs. In the TUH protocol, the planning target volume (PTV) is created by adding a margin of 7 mm to CTV in all other directions except towards the rectum, where a 5 mm margin is used. In SBRT, however, the PTV consists of CTV with an added margin of 5 mm applied in all other directions except towards the rectum, where a 3 mm margin is used [23].

The manual contouring process on MRI followed the ESTRO-ACROP guidelines [34]. The correspondence between all MRI series was confirmed based on implanted markers to account for any intra-fraction motion that may have occurred during the 13-min MRI image acquisition for RTP (26-min including additional sequences for diagnostic use). The mean (SD) times for manual delineations on CT and MRI images were 27 (12) min and 26 (7) min, respectively [10,25]. When the contouring processes were finalized and approved by the ROs, the structure sets from the approved sessions were saved, and the studies were exported to the RTP system.

***Treatment planning***

Prostate cancer patients in both RT workflows were planned according to applied routine clinical practice receiving either 74 (78) Gy in 37 (39) fractions or to 60 Gy in 20 fractions or to 36.25 Gy in 5 fractions. Volumetric-modulated arc therapy treatment plans with two co-planar arcs were optimized and calculated by planners using the Eclipse™ RTP system with an anisotropic analytical algorithm (AAA). The voxel grid size for optimization and dose calculation was set to 2.0 × 2.0 × 2.0 mm^3^ (1.0 × 1.0 × 1.0 mm^3^ in SBRT). The treatment technique was identical in both workflows, as well as was the criteria for target dose coverage and dose uniformity.

In the MRI-only workflow, the RTP including dose calculation was based on sCTs generated by the commercial software (magnetic resonance for calculating attenuation, MRCAT, Philips Oy, Vantaa, Finland) delivered by the manufacturer [35]. Two different versions of the MRCAT were used: the first version used a bulk assignment of Hounsfield unit (HU) values [7], and the second version was an improved version providing continuous HU values [8]. It has been shown earlier that the mean (SD) dose difference in the PTV mean dose is < 0.2% (0.4%) between sCT and CT, and the average minimum gamma-index pass-rates are better than 95% with 2% / 2 mm gamma-criteria [8]. One may note that in prostate SBRT, as well as in prostate RT with MRI contraindications, the RTP and IG are always based on the use of planning CT image set.

***On-board image guidance and treatment delivery***

15-min appointments were scheduled for patients in the treatment units. Treatments were delivered by RTTs using daily IG for the visualization and localization of fiducial markers implanted into the prostate. The 2D kV-radiographs and 3D CBCT images were acquired with the on-board IG systems integrated to linear accelerators (TrueBeam and True Beam STx, Varian Medical Systems Inc., Palo Alto, CA, USA). On the treatment consoles, the patients were setup by matching either 2D or 3D image pairs. For the first three RT fractions, the IG was performed using the pair of 3D sCT/CT and 3D CBCT images. These images were also used for the verification of the target/PTV position and the assessment of rectum and bladder filling. From the fourth RT fraction onwards, the IG was carried out by the marker-based pair of 2D kV-radiographs. In case of difficulties, however, the 3D IG was performed also in later fractions during the course of RT. In SBRT, patient positioning is always based on the use of 3D IG and in addition for the verification of target position, 2D kV-radiographs are acquired at 10-s intervals during the RT delivery.

Setup corrections were always applied in all three (x, y, z) directions without any action level for marker deviations. For 3D IG, the CBCT images were acquired using 125 kV and 80 mAs with a 1.0 × 1.0 mm^2^ in-plane resolution, 2-mm-thick slices and 160 mm coverage. For 2D IG, two orthogonal kV-radiographs were obtained with a pixel size of 0.39 × 0.39 mm^2^ and a field-of-view of 30.0 × 40.0 cm^2^. Usually, the 2D IG was based on orthogonal images but in case of e.g. hip prostheses, there was a possibility to use oblique digitally reconstructed radiographs (DRR) together with oblique kV-radiographs using the ExacTrac (Brainlab AG, Munich, Germany) system for the matching of images. It has been shown earlier that when used, the difference between synthetic DRR- and DRR-based patient positioning is < 0.3 (1.4) mm in all directions, and the matching of sCT to CBCT produces similar results to that of CT to CBCT [8].

***Follow-up after curative radiotherapy***

A single follow-up visit to RO is scheduled three months after the end of the RT course. Then, annual laboratory follow-up of serum prostate specific antigen (PSA) is arranged over a period of the first 10 years. Most patients now agree to a follow-up schema using an automated remote tracking of serum PSA, where an alarm limit is set to the PSA nadir + 2. The patients will be called to a physical visit in hospital only if a PSA alarm value is detected or they report urinary or bowel symptoms, which potentially need a medical intervention. The follow-up visits are identical in both RT workflows.

***References***

[7] Kemppainen R., Suilamo S., Tuokkola T., Lindholm P., Deppe M.H., Keyriläinen J. Magnetic

resonance-only simulation and dose calculation in external beam radiation therapy: a feasibility study for pelvic cancers. Acta Oncol 2017;56:792–798. doi:10.1080/0284186X.2017.1293290.

[8] Kemppainen R., Suilamo S., Ranta I., Pesola M., Halkola A., Eufemio A., et al. Assessment of

dosimetric and positioning accuracy of a magnetic resonance imaging-only solution for external beam radiotherapy of pelvic anatomy. Phys Imaging Radiat Oncol 2019;11:1–8.

doi:10.1016/j.phro.2019.06.001.

[9] Ranta I., Kemppainen R., Keyriläinen J., Suilamo S., Heikkinen S., Kapanen M., et al. Quality assurance measurements of geometric accuracy for magnetic resonance imaging-based radiotherapy

treatment planning. Phys Med 2019;62:47–52. doi:10.1016/j.ejmp.2019.04.022.

[10] Kuisma A., Ranta I., Keyriläinen J., Suilamo S., Wright P., Pesola M., et al. Validation of automated magnetic resonance image segmentation for radiation therapy planning in prostate cancer. Phys Imaging Radiat Oncol 2020;13:14–20. doi:10.1016/j.phro.2020.02.004.

[23] Zilli T., Jorcano S., Bral S., Rubio C., Bruynzeel A.M.E., Oliveira A., et al. Once-a-week or everyother-day urethra-sparing prostate cancer stereotactic body radiotherapy, a randomized phase II trial: 18 months follow-up results. Cancer Med 2020;9:3097–3106. doi:10.1002/cam4.2966.

[25] Kiljunen T., Akram S., Niemelä J., Löyttyniemi E., Seppälä J., Heikkilä J., et al. A deep learning -based automated CT segmentation of prostate cancer anatomy for radiation therapy planning - a retrospective multicenter study. Diagnostics 2020;10:959. doi:10.3390/diagnostics10110959

[34] Mottet N., Bellmunt J., Bolla M., Briers E., Cumberbatch M.G., De Santis M., et al. EAU-ESTROSIOG guidelines on prostate cancer. Part 1: screening, diagnosis, and local treatment with curative intent. Eur Urol 2017;71:618–629. doi:10.1016/j.eururo.2016.08.003.

[35] Köhler M., Vaara T., Van Grootel M., Hoogeveen R., Kemppainen R., Renisch S. MR-only simulation for radiotherapy planning. White paper: Philips MRCAT for prostate dose calculations using only MRI data. Amsterdam: Koninklijke Philips NV; 2015.
